# Supplementary material for: Decreased Levels of Soluble Developmental Endothelial Locus-1 Are Associated with Thrombotic Microangiopathy in Pregnancy
Source: Int J Mol Sci. 2023 Jul 21;24(14):11762. doi: 10.3390/ijms241411762 (PMC10380227; doi:10.3390/ijms241411762)
Supplement: Supplementary file 1 [file ijms-24-11762-s001.zip › ijms-2499029-supplementary.pdf]

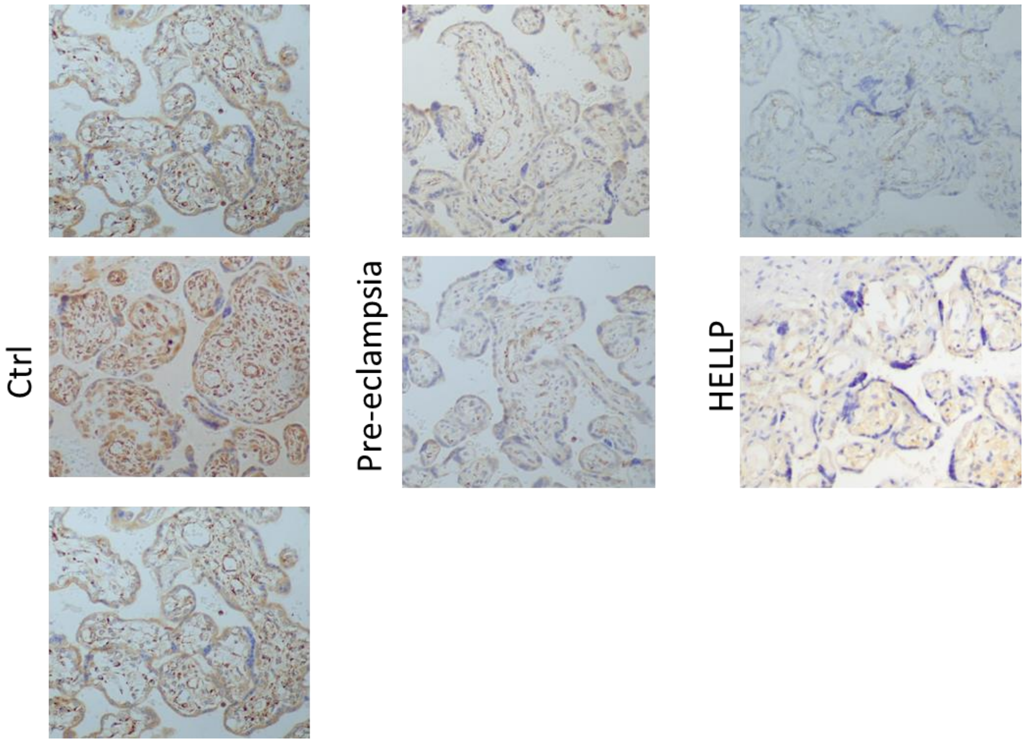

Supplementary Figure S1. Expression of DEL-1 in tissue sections from additionally three women with uncomplicated pregnancies assessed by immuno-histochemistry and two women with preeclampsia and two patients with HELLP syndrome. Magnification 200x
